# Dolutegravir based therapy showed CD4^+^ T cell count recovery and viral load suppression among ART naïve people living with HIV AIDS: a pilot evaluation

**DOI:** 10.1038/s41598-024-53282-y

**Published:** 2024-02-08

**Authors:** Teshager Gebremedhin, Melak Aynalem, Mohammed Adem, Demeke Geremew, Yetemwork Aleka, Amare Kiflie

**Affiliations:** 1 University of Gondar Comprehensive Specialized Hospital, Gondar, Ethiopia; 2 Department of Immunology and Molecular Biology, University of Gondar, Gondar, Ethiopia; 3 Department of Medical Laboratory Sciences, Immunology and Molecular Biology Unit, College of Medicine and Health Sciences, Bahir Dar University, Bahir Dar, Ethiopia; 4 Department of Hematology and Immunohematology, University of Gondar, Gondar, Ethiopia

**Keywords:** Immunology, Molecular biology

## Abstract

Recently, dolutegravir (DTG)-based combined therapy, a more effective and safer first-line antiretroviral therapy (ART), has been recommended by the World Health Organization for the treatment of Human Immunodeficiency Virus (HIV) since July 2018. However, its effectiveness in CD4^+^ T-cells count recovery and viral load suppression has not been studied yet in Ethiopia, where HIV is endemic. Therefore, we aimed to conduct a pilot assessment on the effect of DTG-based therapy on CD4^+^ T-cell count and viral load count among people living with HIV (PLWH) in Ethiopia. A longitudinal prospective cohort study was conducted from July 2020 to February 2021. 109 PLWH who are ART naive but plan to initiate DTG-based therapy were recruited. HIV viral ribonucleic acid (RNA) copies were determined using polymerase chain reaction. To compute the difference in viral load and CD4^+^ T-cell counts between the baseline, 3rd, and 6th months, a Friedman test was used. The study included 109 PLWH who had never received antiretroviral medication. Participants taking DTG-based treatment showed significantly decreasing median (IQR) values of viral load count (copies/mL) from 446,812 (237649.5–732994.5) at baseline to 34 (23.5–46) at 3 months and 0.0 (0–19) at 6 months of treatment follow-up. Although the treatment increases the proportion of participants with HIV-1 RNA 50 copies/mL from 0 (0% at baseline) to 87 (79.8%) and 100 (91.7%) at the 3rd and 6th months of treatment, respectively, On the other hand, the CD4^+^ T-cell count increased significantly during treatment: median (IQR): 209 (81.5–417.5) versus 291 (132–522) versus 378 (181–632.5) cells/L at baseline, the 3rd and 6th months of the treatment follow-up period, respectively. We found dolutegravir-based therapy was a promising option with high virological suppression rates and CD4^+^ T-cell count recovery, demonstrating a restoration of cellular immunity. Moreover, Viral load suppression rates were high after the initiation of the treatment. We recommend further research should be conducted with a larger number of participants to acquire greater awareness of the treatment outcomes.

## Introduction

Effectiveness, safety, and durability of the antiretroviral treatment regimen have always been important factors in Human Immunodeficiency (HIV) chronic care. Recent developments in antiretroviral therapy have demonstrated the development of more effective and secure regimens^1^.

According to the most recent World Health Organization (WHO) guidelines, a combination of at least three antiretroviral therapies (cART) is required for the management of chronic human immunodeficiency virus (HIV)^2^. Two nucleoside reverse transcriptase inhibitors with a non-nucleoside reverse transcriptase inhibitor make up the initial line of ART that is recommended for adults. The Dolutegravir (DTG) + Tenofovir (TDF) + Lamivudine (3TC)-based regimen is recommended by the World Health Organization as the first-line antiretroviral therapy for patients with HIV^2^. These regimens outperform conventional treatment regimens in both treatment-naive and treatment-experienced individuals, including those who have previously failed raltegravir or elvitegravir^3^.

In the United States and Europe, dolutegravir-based antiretroviral therapy has been approved for first-line HIV treatment. Around 70 low- and middle-income countries planned to incorporate DTG into their national recommendations by the end of 2017, shifting to a DTG-based first-line regimen as recommended by WHO^2^.

However, extensive analysis of several important populations, such as those with different genetic diversity, pregnant women, those with HIV-tuberculosis co-infection receiving rifampicin-based treatments, and patients from developing nations who had previously received treatment but were treatment-naive or drug-resistant to nucleoside reverse transcriptase inhibitors, was not included in the development programs for DTG-based regimens^4,5^. The effectiveness and safety of DTG-based therapy have other limitations. Therefore, there is an urgent need for additional populations with relevance to public health and for supplementary investigations^6^.

Despite the fact that different treatment efficacy studies have revealed that the drug regimen dolutegravir antiretroviral therapy promotes immune system recovery^1,7–9^, no longitudinal studies have been conducted in Ethiopia because viral strain types differ and the number of clients on second-line ART has increased in recent years. A pilot study, for example, found that dolutegravir (DTG) + lamivudine (3TC) participants developed resistance mutations. While new DTG-based regimens must be tested for efficacy if health system factors related to the emergence of drug resistance has to be minimized^10–12^.

Early ART initiation once an HIV diagnosis is established plays a significant role in lowering mortality and morbidity due to HIV. Therefore, the goal of this study is to provide further light on the effectiveness of DTG-based regimens and their function in immunological recovery and viral load suppression, especially in Ethiopian settings. It will also serve as a starting point for scaling up the treatment in the future for the purposes of viral suppression and immunologic advantages.

## Methods

### Study area

The study was conducted at ART clinics of Gondar town health facilities. Currently, University of Gondar (UoG) comprehensive specialized referral hospital, Teda, Azezo, Maraki, Gondar Health centers, Haset clinic, and Ibex Hospital who provide services related to ART.

### Study design and population

A longitudinal prospective cohort study was performed from July 2020 to February 2021 at Gondar town health facilities of ART clinic with a continuing enrolment of ART naïve PLWH individuals who initiated ART containing TDF + 3TC (FTC) + DTG among ART naïve People living with HIV AIDS individuals in Gondar town, Northwest Ethiopia. Socio-demographic data were collected using a structured questionnaire while clinical data was collected from patient record forms from all study participants,

### Operational definitions

A good immunological response is > 20% increase in CD4^+^ T-cells count from baseline during the first the 6th month of ART whereas Immunologic failure is defined as having a CD4^+^ T-cell count result < 20% cells/µL at the end of the 6th month^13^.

Viral suppression is defined as proportion of patients achieving plasma viral load suppression < 50 copies/mL within the 3rd and 6th months of DTG based regimen initiation. Virologic failure is defined as the proportion of patients achieving plasma viral load suppression > 50 copies/mL within the 3rd and 6th months of DTG based regimen initiation^14^.

Good ART adherence—equal to or greater than 95% adherence i.e., missing only 1 out of 30 doses or missing 2 from the 60 doses implies good adherence. Fair ART adherence—85–94% adherence, i.e., missing 2–4 doses out of 30 doses or 4 to 9 doses from 60 doses. Poor ART adherence—less than 85% adherence, i.e., missing > 5 doses out of 30 doses or > 10 doses from 60 doses implies poor adherence^15^. BMI levels < 18.5, 18.5–24.9, 25.0–29.9, 30.0, and above are underweight, normal, overweight, and obese, respectively^16^.

### Blood sample collection

For CD4^+^ T-cell and viral load counts, eight mL of venous blood was collected into two separate EDTA vacutainer tubes. On the same day as sample collection, whole blood and plasma samples were transported to the UOG comprehensive specialized hospital ART clinic. One tube containing 5 ml of whole blood was centrifuged at 1500 RPM for 10 min, and plasma was separated for HIV-1 viral load testing at the site of sample collection while the other tube remained at room temperature. All tests were carried out in accordance with the relevant laws and regulations of the World Medical Association (WMA) declaration of Helsinki. Data and samples were collected as part of the standard Anti -Retroviral Therapy (ART) provided by the University of Gondar's ART clinic.

### Flowcytometry and TaqMan nucleic acid amplification assay

A CD4^+^ T helper cell count was performed using the BD FACSPresto Near-Patient CD4 Counter System. A whole blood sample was stained using fluorochrome-conjugated antibodies (CD4 PE-Cy5, CD3 APC, and CD45RA APC dried antibody reagents), and when the specific antibodies bind to the surface antigens on the T lymphocytes during the incubation period, dedicated software identifies and counts the CD4^+^ T lymphocyte^17^.

HIV-1 viral load was performed from a stored plasma sample following an appropriate thawing procedure using the COBAS Ampliprep/COBAS TaqMan nucleic acid amplification test for the quantification of HIV-1 RNA in plasma^18^.

### Data analysis and processing

The data was entered and cleared by EPI INFO 7, exported, and analyzed using GraphPad prism V.5.03 (GraphPad Software, USA) comparisons between baseline, 3rd, and 6th months of HIV viral load, and CD4^+^ T-cell counts were performed by using Friedman test. A continuous variable was expressed as median, interquartile range (IQR), and proportion or percentage. All *P* values < 0.05 were considered statistically significant.

### Ethical consideration

The study was approved by the School of Biomedical and laboratory science ethical review committee, University of Gondar. Permission letter was taken from the University of Gondar comprehensive and specialized referral hospital and other health facilities. The objective of this research was explained to the study participants and written informed consent was obtained from all the study participants. Those willing to participate were enrolled. Data was used for research and publication purposes only and access to the data was restricted to the researcher, supervisors, and statistician. No client names were collected but unique identifiers were used. The data used in this study were stored securely. Any abnormal result was immediately given to their physicians.

## Results

### Socio-demographic and clinical characteristics

We followed 109 study subjects longitudinally. Fifty-six (51.4%) of them were male with a median age of 32 (IQR: 26.5–40) and 94 (86.2%) were living in urban. At baseline, 38 (34.9%) and 31 (28.4%) of study participants were in WHO clinical stage I and III, respectively. Besides, 86 (78.9%) & 84 (77.1%) of the study participants had good ART adherence during the 3rd & 6th months of follow-up, respectively (Table 1).

**Table 1:** Socio-demographic and clinical characteristics of the study participants attending ART clinics at Gondar, Northwest Ethiopia.

Variables	N (%)
Age, median (range)	32 (18–56)
Sex	
Male	56 (51.4%)
Female	53 (48.6%)
Residence	
Urban	94 (86.2%)
Rural	15 (13.8%)
WHO clinical stage	
Stage I	38 (34.9%)
Stage II	24 (22.0%)
Stage III	31 (28.4%)
Stage IV	16 (14.7%)
3-month ART adherences	
Good	86 (78.9%)
Fair	10 (9.2%)
Poor	13 (11.9%)
6-month ART adherences	
Good	84 (77.1%)
Fair	10 (9.2%)
Poor	15 (13.8%)
BMI categories	
< 18.5	35 (32.1%)
18.5–24.9	65 (59.6%)
25–29.9	8 (7.3%)
> 30	1 (.9%)

### Virological efficacy

Of 109 participants, the median baseline, 3rd, and 6th months of HIV viral load count was 446,812 (IQR: 237650–732995), 34 (IQR: 23.5–46), 0.0 (IQR: 0–19), copies/mL, respectively. At the 3rd and 6th months of follow-up, the proportion of participants with HIV-1 RNA < 50 copies/mL were 87 (79.8%) and 100 (91.7%) of 109 participants, respectively (Table S1) (supplementary).

### Dolutegravir based therapy and viral load

**Figure 1:**
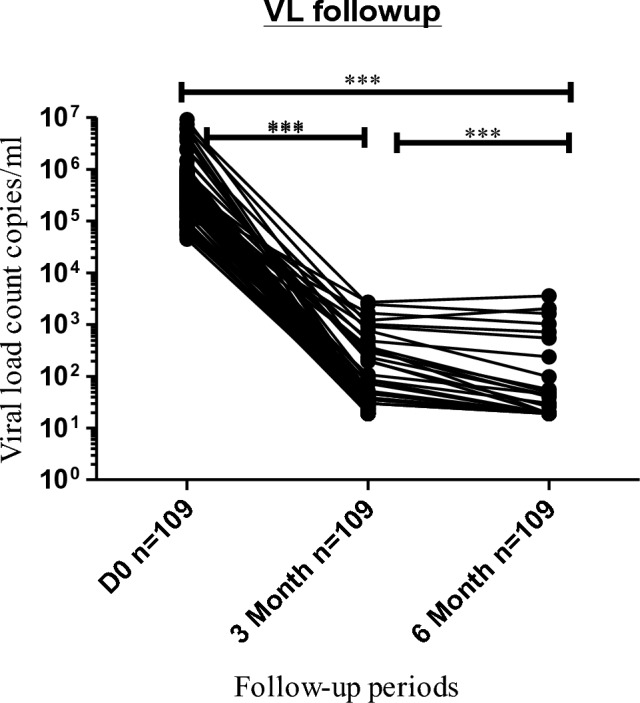
Effect of dolutegravir based therapy on viral load counts among ART naïve participants in Gondar, Ethiopia. The above line graph indicates the viral load suppression from baseline according to antiretroviral regimens at different periods of treatment. A Friedman test was used to compute the difference in viral load counts between the baseline, 3rd, and 6th months. *Statistically significant at *P* < 0.05, **statistically significant at *P* < 0.01, ***statistically significant at *P* < 0.001.

There was significant viral suppression observed between baseline, 3rd, and 6th months. The median (IQR) viral RNA copies per ml were 446,812 (IQR: 237650–732995) at baseline while it becomes 34 (IQR: 23.5–46), and 0.0 (IQR: 0–19) at 3rd and 6 months of treatment follow-up periods, respectively (*P* < 0.001) (Fig. 1).

### Dolutegravir based therapy has a significant effect on viral load count across the BMI categories during follow-up periods

**Figure 2:**
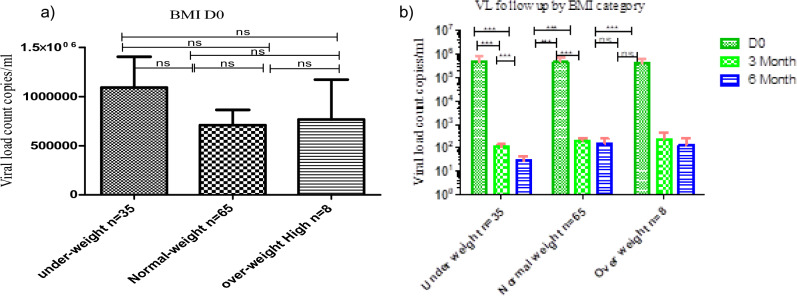
Dolutegravir based therapy has a significant effect on viral load across the BMI categories among treatment naïve individuals in Gondar, Ethiopia. Histogram (**a**) showing the baseline differences of viral load count across BMI categories and Histogram (**b**) showing the rate of viral load suppression (copies/ml/months) by BMI categories between the different periods following ART. Friedman test was used to compute the difference in viral load counts between the baseline, 3rd, and 6th months of follow-up period under the BMI categories. *Statistically significant at *P* < 0.05, **statistically significant at *P* < 0.01, ***statistically significant at *P* < 0.001.

There was no significant difference comparing the median viral load count across all the BMI categories under-weight, normal, overweight, and obese individuals during baseline (Fig. 2a). However, we observed statically significant viral load suppression during follow-up periods (Between D0, 3rd and 6th months) compared across BMI categories (*P* < 0.001) (Fig. 2b).

### Dolutegravir based therapy effect across WHO clinical stage

**Figure 3:**
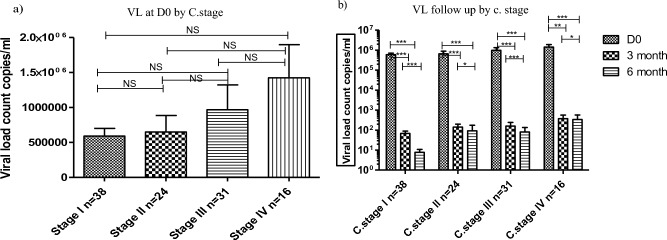
Dolutegravir based therapy has a significant effect on viral load across WHO clinical stage among PLWH individuals in Gondar, Ethiopia. Histogram (**a**) showing the baseline differences of viral load count with WHO C. Stage of HV. And Histogram (**b**) showing the rate of viral load suppression (copies/ml/months) under-WHO clinical stage of HIV between the different periods following ART. Friedman test was used to compute the difference in viral load counts between the baseline, 3rd, and 6th months of follow-up period under WHO clinical stage. *Statistically significant at *P* < 0.05, **statistically significant at *P* < 0.01, ***statistically significant at *P* < 0.001.

Comparing the viral load count between the baseline with 3rd, and 6th months under-WHO clinical stage I (*P* < 0.001), stage II (*P* < 0.001), and stage III (*P* < 0.001) showed a significant difference (Fig. 3a). We also observed high virological suppression in the comparison of baseline with 3rd (*P* < 0.01), and 6th (*P* < 0.001) months of follow-up in those patients under WHO stage IV presented (Fig. 3b).

### Good ART adherence showed a significant viral load suppression during treatment periods

Study participants who had good adherence had shown association with viral load suppression (*P* = 0.000) while study participants with fair adherence did not show significant association with viral load counts (*P* = 0.625) (Table 2).

**Table 2:** Association of viral load with adherence of study subjects attending ART clinics at Gondar, Northwest Ethiopia.

Independent variables	COR	95% CI	*P* value
Three months adherence			
Good	0.073	0.020–0.272	0.000
Fair	0.625	0.118–3.316	0.581
Poor	1		
Six months adherence			
Good	0.016	0.002–0.149	0.000
Fair	0.333	0.051–2.177	0.333
Poor	1		

### CD4^+^ T-cell recovery

**Figure 4:**
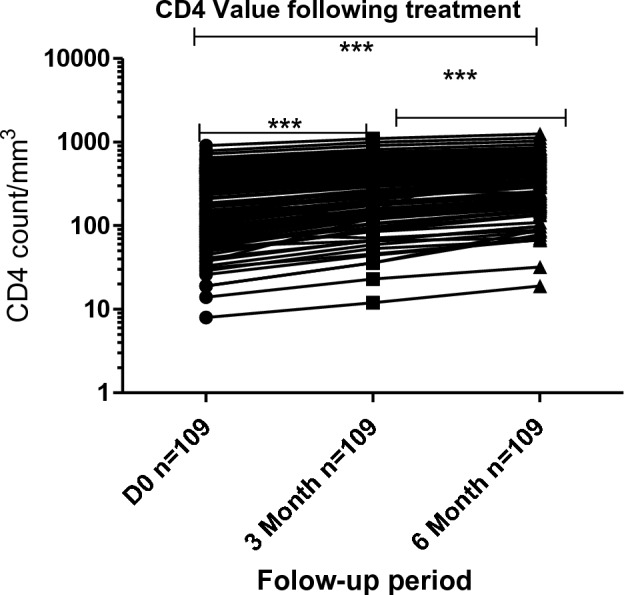
Effect of dolutegravir based therapy on CD4^+^ T cell counts. The above line graph indicates the CD4^+^ T-cell count change from baseline according to antiretroviral regimens at different periods of treatment. Friedman test was used to compute the difference in CD4^+^ T-cell counts between the baseline, 3rd, and 6th months of follow-up period. *Statistically significant at *P* < 0.05, **statistically significant at *P* < 0.01, ***statistically significant at *P* < 0.001.

Of 109 participants, the median baseline, 3rd, and 6th months of CD4^+^ T-cell count were 209 (IQR: 81.5–417.5), 291 (IQR: 132–522), 378 (IQR: 181.-632.5), cells/µL, respectively. Among WHO clinical stage one, the median baseline, 3rd, and 6th months of CD4^+^ T-cell count was 370.5 (IQR: 229–480.8), 501.5 (IQR: 317.8–634), 571 (IQR: 391.8–747.5), cells/µL, respectively (Table S2) (supplementary data). A good CD4^+^ T-cell counts recovery was observed between baseline and 3rd, and 6th months of follow up periods (*P* < 0.001) (Fig. 4).

### Dolutegravir based therapy has a significant effect on CD4^+^ T-cell count across the BMI categories

**Figure 5:**
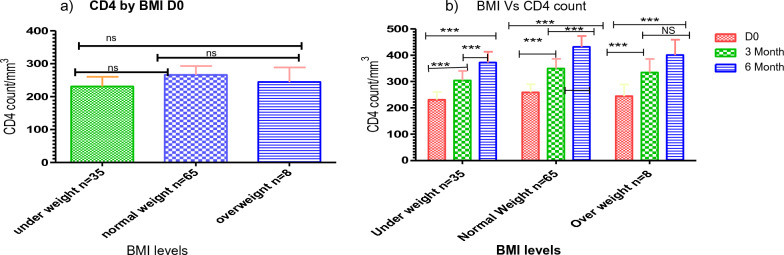
Dolutegravir based therapy has a significant effect on CD4^+^ T-cell count across the BMI categories. Histogram (**a**) showing the baseline differences of CD4^+^—T cell count across the BMI categories and Histogram (**b**) showing the rate of CD4^+^ T-cell count increased (cells/µL/months) by BMI group between the different periods of ART follow-up. Friedman test was used to compute the difference in CD4^+^ T-cell counts between the baseline, 3rd, and 6th months of follow-up period. *Statistically significant at *P* < 0.05, **statistically significant at *P* < 0.01, ***statistically significant at *P* < 0.001.

Similarly, with viral load count, there was no significant association in CD4^+^ T-cell counts at base line comparing across all BMI categories (Fig. 5a). In contrast, a good CD4^+^ T-cell counts recovery was observed between baseline and 6th months follow-up of CD4^+^ T-cell count across all the BMI categories (*P* < 0.001) (Fig. 5b).

### CD4^+^ T-cell count recovery across WHO clinical stage

**Figure 6:**
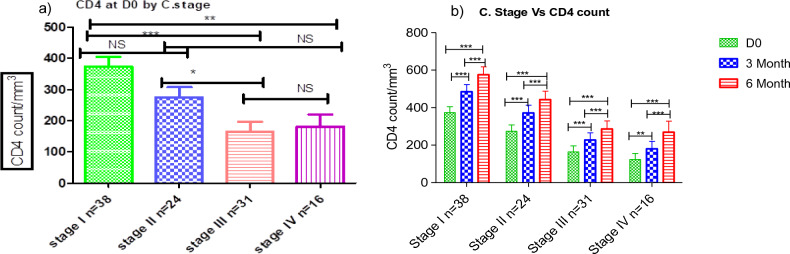
Dolutegravir based therapy has a significant effect on CD4^+^ T-cell count across WHO clinical stage. Histogram (**a**) showing the baseline differences of CD4^+^ T- cell count across WHO C. Stage of HV and Histogram (**b**) showing the rate of CD4^+^ T-cell count increased (cells/µL/months) by WHO clinical stage of HIV between the different periods following ART. Friedman test was used to compute the difference in CD4^+^ T-cell counts between the baseline, 3rd, and 6th months of follow-up period. *Statistically significant at *P* < 0.05, **statistically significant at *P* < 0.01, ***statistically significant at *P* < 0.001.

We found a significant CD4^+^ T-cell count difference during treatment initiation period between clinical stage I and IV (Fig. 6a). Moreover, a decent CD4^+^ T-cell counts changing was observed between 3rd and 6th months follow-up treatment of CD4^+^ T-cell count under-all WHO clinical stage (*P* < 0.001) (Fig. 6b).

## Discussion

Dolutegravir based regimen therapy is an efficient, well-tolerated drug, and associated with decreasing viral RNA copies^19^. As revealed by previous studies^1,8,20–22^, 91.7% individuals with plasma HIV RNA less than 50 copies/mL is increased sharply from baseline to 6th months. The proportion of participants achieving plasma HIV-1 RNA less than 50 copies/mL were 79.8% (95% CI 71.6–87.2), and 91.7% (95% CI 86.2–96.3) in the 3rd and 6th months, respectively. There was a statistically significant difference in viral load suppression between baseline and 3rd and 6th months of follow-up (*P* < 0.001). These results highlight the tremendous accomplishments of the widespread implementation of the treatment, which has led to significant health benefits and an increase in life expectancy^22^.

In contrast to the Italian investigation, which revealed that 81% of individuals had viral suppression of 50 copies/mL during the sixth months of follow-up, we discovered lower levels of viral RNA copies^21^. In a related study done in French, it was discovered that 3 months after starting a dolutegravir-based treatment, 58% of participants had viral suppression that was fewer than 50 copies/mL^12^. This discrepancy between findings may be caused by differences in geographic location or genetic make-up.

The DTG-based regimen showed a rise in CD4^+^ T-cell count in patients with significant viremia, demonstrating its efficacy in naive individuals. In addition, we demonstrated that the median CD4^+^ T-cell count at baseline was 209 (IQR: 81.5–417.5), cells/L, corresponding to a 28 cells/L monthly recovery rate. This was also comparable with a Brazilian survey^22^.

During follow-ups at the third and sixth months of DTG-based regimen treatment, higher median CD4^+^ T-cell counts were seen in the current study. The median CD4^+^ T-cell count at baseline was 209 cells/L (IQR: 81.5–417.5), and at the third and sixth months, it was 291 cells/L (IQR: 132–522) and 378 cells/L (IQR: 181.-632.5), respectively (*P* 0.001). Comparably, a study done in Germany showed that, based on a median CD4^+^ T-cell count of 258, the obtained cells at the third and sixth months were 345 and 465 cells/L, respectively^1^.

Similar findings were found in a study on dolutegravir-based therapy carried out in the United States, which showed that the median (interquartile range) increase in CD4^+^ T-cell counts from baseline at six months was 167 (86, 275) cells/L^23^.

Furthermore, severe immune suppression may continue in some patients, particularly those who began ART with a very low CD4 cell count and who do not experience a significant improvement in their CD4 cell count after treatment^24^. However, like the study conducted in Kenya^25^, the median CD4^+^ T-cell counts changing across all WHO clinical stages in our study clearly exhibited statistical significance (*P* 0.001).

A WHO recommendation states that sustained high levels of adherence are necessary to stop viral replication and reduce the risk of developing antiretroviral treatment resistance^2^. Additionally, we found a significant association between viral load and ART adherence (*P* = 0.000). We also found that poor ART adherence had predictors of first-line ART treatment failure (*P* = 0.000). Research conducted in Kenya and Ethiopia provide additional evidence in favor of this^24,25^.

Likewise, the absence of viral suppression along with immunologic, clinical, or other deterioration is a symptom of therapeutic failure^6^. It is interesting to note that between baseline and the third and sixth months of follow-up, the viral load reduced statistically significantly across all WHO clinical stages. The studies carried out in Kenya and Ethiopia^24^ offered additional proof of this^25^. Also, research undertaken in Switzerland and the Island revealed a correlation between the WHO clinical stage and treatment failure^24,26^.

Low body mass, weight loss, and HIV disease progression are all independent risk factors for mortality, according to WHO guidelines. Therefore, nutritional supplements may be necessary for HIV-infected individuals who are malnourished in addition to ART to aid in their nutritional recovery^2^. In our investigation, we discovered a statistically significant difference in viral load suppression between baseline, third, and sixth months of treatment with all BMI levels (*P* 0.001). Likewise, According to studies done in the United States, China, and Island, HIV-infected people who are overweight or obese had lower viral load levels than those who are normal weight or have low baseline BMI^24,27^. Since there was only one obese individual in our study and Leptin may play a role in the mechanism connecting fat and overweight to immunological function in HIV-infected people, which may account for the difference. On the contrary, we only recruited 109 study participants, and the lower rate of obesity may be due to the small sample size. An adipocyte-derived hormone that affects body weight is leptin. Serum leptin levels have been discovered to be greater in overweight and obese people than in people of normal weight^27^.

## Conclusion and recommendation

The findings of our study clearly demonstrate that for HIV-infected people who have never received treatment, dolutegravir-based therapy is a viable treatment option with high rates of virological suppression and a median change in CD4^+^ T-cell count. Viral load suppression rates were high among ART patients. We suggest that additional research including a large participant pool associated with various underline clinical condition and comparison analysis needs to be conducted.

### Supplementary Information

Supplementary Tables.

## Data Availability

The data used to support the findings of this study are restricted by the research ethics committee of the school of Biomedical and Laboratory Sciences, College of Medicine and Health Sciences, University of Gondar. Data are available from Teshager Gebremedhin for researchers who meet the criteria for access to confidential data.

## Abbreviations

3TC: Lamivudine
AIDS: Acquired Immune Deficiency Syndrome
ART: Antiretroviral therapy
BD FACS: Becton Dickinson fluorescence activated cell sorting
BMI: Body mass index
cART: Combination antiretroviral therapy
CD: Cluster of differentiation
DTG: Dolutegravir
D0: Day zero
EDTA: Ethylene diamine tetra-acetic acid
EFV: Efavirenz
FTC: Emtricitabine
HAART: Highly active antiretroviral therapy
HIV: Human Immunodeficiency Virus
ML: Milliliter
PCR: Polymerase chain reaction
PLWH: People living with HIV
PMTCT: Prevention of mother-to-child transmission
QS: Quantification standard
RNA: Ribose nucleic acid
SPU: Sample processing unit
TDF: Tenofovir disoproxil fumarate
UNAIDS: United Nations Program on HIV/AIDS
UoG: University of Gondar
WHO: World Health Organization
