# Supplementary material for: Dolutegravir based therapy showed CD4+ T cell count recovery and viral load suppression among ART naïve people living with HIV AIDS: a pilot evaluation
Source: Sci Rep. 2024 Feb 8;14:3297. doi: 10.1038/s41598-024-53282-y (PMC10853173; doi:10.1038/s41598-024-53282-y)
Supplement: Supplementary file 1 — Supplementary Tables. [file 41598_2024_53282_MOESM1_ESM.docx]

Table S1: High Virological suppression during comparing viral load with BMI categories and HIV clinical Stage of study subjects attending ART clinics at Gondar, North west Ethiopia.

| ***Variables*** | ***Category*** | | | ***Baseline*** | ***3 months*** | ***6 months*** |
| --- | --- | --- | --- | --- | --- | --- |
| ***HIV viral load count*** | | *median, copies/ml* | | 446812 | 34 | 0.0 |
|  |  | *IQR, copies/ml* | | 237649.5-732994.5 | 23.5-46 | 0-19 |
|  |  | *<50 copies/ml, n (%)* | | 0 (0%) | 87 (79.8%) | 100(91.7%) |
|  |  | *<50 copies/ml, 95% confidence interval lower, upper* | | 0 | 71.6-87.2 | 86.2-96.3 |
|  |  | *>50 copies/ml, n (%)* | | 109 (100%) | 22 (20.2%)) | 9 (8.3%) |
|  |  | *>50 copies/ml ,95% confidence interval lower, upper* | | 109 | 12.8-28.4 | 3.7-13.8 |
| ***Viral load VS BMI categories*** | | *Under-weight n=35* | *median, copies/ml* | 474392 | 32 | 0.0 |
|  |  |  | *IQR, copies/ml* | 266187- 834521 | 27- 46 | 0.0-0.0 |
|  |  | *Normal weight n=65* | *median, copies/ml* | 434563 | 35.00 | 0.0 |
|  |  |  | *IQR, copies/ml* | 200291- 672194 | 21- 46 | 0.0- 19 |
|  |  | *Over-weight n=8* | *median, copies/ml* | 423658 | 36 | 0.0 |
|  |  |  | *IQR, copies/ml* | 175836- 650217 | 19.25- 76 | 0.0- 19 |
|  |  | *Obese n=1* | *median, copies/ml* | 356224 | 43 | 0.0 |
|  |  |  | *IQR, copies/ml* | 356224 | 43.00 | 0.0 |
| ***viral load vs HIV C. Stage*** | | *Stage I n=38* | *median, copies/ml* | 425483 | 31.50 | 0.0 |
|  |  |  | *IQR, copies/ml* | 204185- 682885 | 19.75- 41.75 | 0.0 -4.750 |
|  |  | *Stage II n= 24* | *median, copies/ml* | 444108 | 38.50 | 0.0 |
|  |  |  | *IQR, copies/ml* | 200774- 677897 | 23.75- 66.25 | 0.0 -14.25 |
|  |  | *Stage III n=31* | *median, copies/ml* | 435296 | 32.00 | 0.0 |
|  |  |  | *IQR, copies/ml* | 242985- 767281 | 24- 46 | 0.0- 0.0 |
|  |  | *Stage IV n=16* | *median, copies/ml* | 493967 | 35 | 0.0 |
|  |  |  | *IQR, copies/ml* | 256850-2.127e+006 | 24.75- 102.5 | 0.0- 41.25 |

*n= Number IQR=Inter Quartile Range*

**Table S2:** A decent CD4^+^ T-cell count changing during comparing CD4^+^ T-cell with BMI categories and HIV clinical Stage of study participants attending ART clinics at Gondar, North west Ethiopia.

| ***Variable*** | *Category* | | Baseline | 3 months | 6 months |
| --- | --- | --- | --- | --- | --- |
| ***CD4^+^ T-cell count*** | *median, cells/µL* | | 209 | 291 | 378 |
|  | *IQR, cells/µL* | | 81.5-417.5 | 132-522 | 181.-632.5 |
| ***CD4^+^ T-cell count vs BMI categories*** | *Underweight*  *n=35* | *median, cells/µL* | 167 | 227 | 322 |
|  |  | *IQR, cells/µL* | 74- 364 | 106-514 | 148-587 |
|  | *Normal-weight*  *n=65* | *median, cells/µL* | 209.0 | 291 | 378 |
|  |  | *IQR, cells/µL* | 85- 434 | 138.5-560 | 195.5-688.5 |
|  | *Over-weight n=8* | *median, cells/µL* | 200.5 | 285.5 | 366.5 |
|  |  | *IQR, cells/µL* | 172.8- 369.5 | 256-488.8 | 268.8-573.8 |
|  | *Obese n=1* | *median, cells/µL* | 522 | 659 | 738 |
|  |  | *IQR, cells/µL* | 522- 522 | 659 | 738 |
| ***CD4^+^ T-cell count vs HIV C. Stage*** | *Stage I n=38* | *median, cells/µL* | 370.5 | 501.5 | 571 |
|  |  | *IQR, cells/µL* | 229- 480.8 | 317.8-634 | 391.8-747.5 |
|  | *Stage II n=24* | *median, cells/µL* | 272.0 | 370 | 420.5 |
|  |  | *IQR, cells/µL* | 124 363.3 | 188.8-485.3 | 253-581.8 |
|  | *Stage III n=31* | *median, cells/µL* | 85.00 | 132 | 179 |
|  |  | *IQR, cells/µL* | 51- 170 | 86-255 | 133-337 |
|  | *Stage IV n=16* | *median, cells/µL* | 130.5 | 130.5 | 189.0 |
|  |  | *IQR, cells/µL* | 98.25- 251 | 98.25-251 | 147.3-335.5 |

*n= Number IQR=Inter Quartile Range*
